# Influence of Loss Aversion and Income Effect on Consumer Food Choice for Food Safety and Quality Labels

**DOI:** 10.3389/fpsyg.2021.711671

**Published:** 2021-07-08

**Authors:** Wenjing Nie, Huimin Bo, Jing Liu, Taiping Li

**Affiliations:** ^1^College of Humanities and Social Development, Nanjing Agricultural University, Nanjing, China; ^2^College of Economics and Management, Nanjing Agricultural University, Nanjing, China

**Keywords:** food choice, difference, food safety, food quality, loss aversion, income effect

## Abstract

Food safety and food quality are two closely related aspects of the food management system. The difference between the two is that one keeps consumers safe while the other keeps consumers satisfied. This study examined the differences in how consumers value food safety and food quality with a focus on the influence of loss aversion on one’s psychological level and of income effect on one’s socio-demographic level. Our findings indicate that loss aversion and income effect significantly influence the way consumers value food safety vs. quality labels when considering potential health risks and food price. High risk-averse and low-income consumers with strong loss aversion and a weak income effect show a higher demand for food safety labels as a way to ensure easy access to safety indications. Low risk-averse and high-income consumers with weak loss aversion and a strong income effect show a higher demand for food quality labels because they hope to gain more health benefits from high-quality food at good prices. This study provides insights that will assist public authorities and food industry in balancing food safety control and food quality improvement in order to meet the heterogeneous market demand changing alongside the transition of China’s food consumption and production.

## Introduction

Since the food safety scandal of excess melamine residues broke out in China in 2008, the overwhelming majority of consumers with high risk aversion have been in a panic over the issue of food safety (Ortega et al., 2012; Chen et al., 2015; Lai et al., 2018; Liu et al., 2020). Competent Chinese authorities have stepped up food monitoring and supervision along the entire food supply chain from production to consumption (Kang, 2019; Zhou et al., 2021). Since the scandal, food safety risks have been kept under effective control, ensuring that safe food is widely accessible to the public. As incomes rise and the availability of food becomes more diversified, Chinese consumers are beginning to pursue higher quality, more nutritious, and healthier food products (Yu and Abler, 2009; Yu, 2018). In an effort to achieve high-quality improvement in agricultural production, the national rejuvenation initiative has been promoted with the purpose of overhauling the agricultural supply side system and constructing the competitive market order for better quality food to be offered at a good price (Nie et al., 2020; Nie et al., 2021). However, the ongoing COVID-19 pandemic presents an exceptional and unprecedented challenge for the world. Cold-chain food is seen as being one of the major mediums of virus breeding and dissemination, as it carries the virus and raises the risk of infection through food production, processing, storage, packaging, and distribution (Godoy et al., 2021; Han et al., 2021). The outbreak of epidemic disease has a huge impact on public health (Djekic et al., 2021; Marti et al., 2021); thus, food safety issues have resurfaced as a global critical problem along with an increase in consumers’ concern about food safety (Kitz et al., 2021). In fact, consumers’ food safety concerns and food quality demands have clearly transformed their food choices, resulting in changes in the economic structure and food production behavior (Grunert et al., 2015; FAO, 2017). At the current crossroad, whether public authorities and the food industry prioritize food safety control or food quality improvement should be based on the heterogeneous market demand for food safety vs. quality (Choi et al., 2018a, b). After all, consumers with varying levels of risk aversion and income are the primary beneficiaries of the market.

Food safety and food quality are two of the most important aspects of the food management system. Although closely related, there are differences between food safety and food quality regarding the requirements in a food-handling environment (Haas et al., 2021). Food safety refers to practices and conditions that preserve food quality by avoiding contamination and food-borne illnesses during preparation, processing, and storage (Grunert, 2005). Food quality refers to the features and characteristics of a food product that conform to the required specifications, are acceptable and cost-effective to consumers, and are profitable for the company (Naspetti and Zanoli, 2009). The difference between food safety and food quality lies in the fact that one keeps consumers safe and the other keeps consumers satisfied (Rijswijk and Frewer, 2008; Kealesitse and Kabama, 2012). In general, it is difficult for consumers to distinguish the specific attributes of food safety and food quality when making food choices (Dulleck et al., 2011). Food safety is perceived as a credence attributes, as it cannot be ascertained before or after purchase unless through safety indication (e.g., safety certificates, traceable information) (Grunert, 2005). Some quality cues are either search attributes (e.g., brand reputation, quality grades) or credence attributes (e.g., geographical production) (Akdeniz et al., 2013; Grunert et al., 2015; Marek et al., 2020), which can be ascertained prior to consumption (Nelson, 1970; Grunert, 1997). In response to the rising concern over food safety and quality issues as well as differentiated consumer demand for these issues, China has integrated a multilevel food-labeling scheme into the food market in order to help consumers identify the safety and quality levels of food products. The food labeling scheme consists of certification, traceability, brand, grading, geographical indication, etc. Food with certified and traceable labels are primarily consumed out of concern about food safety issue (Nie et al., 2018; Liu et al., 2019; Wu et al., 2020). Brand, quality grade, and geographical indication are introduced to identify the quality levels of food products (Lim and Hu, 2016; Choi et al., 2018b; Ding and Veeman, 2019).

However, much of the previous literature confused the distinction between the concepts of food safety and food quality, and others primarily focused on consumers’ food demand choice for either food safety or food quality. To the best of our knowledge, no published study has integrated food safety and food quality into a comprehensive framework, compared the differences of how consumers value food safety vs. quality labels, and even evaluated how socio-demographic and psychological characteristics influence consumers’ food demand choice. This study addresses the knowledge gap that exists in the current literature of behavioral economics and psychology. Our empirical analysis aims to (1) estimate whether consumers’ food demand choice differs significantly when it comes to food safety vs. quality labels and to compare the extent of those differences in their food choices, and to (2) investigate how loss aversion [i.e., losses loom larger than gains (Koan et al., 2021)] and income effect influence consumers’ food choice for food safety vs. quality labels.

The major objective of this study is to estimate the differences in consumers’ food choice for food safety vs. quality labels. Specifically, we first use the mixed logit model to explore the heterogeneities in consumers’ valuations of food safety vs. quality labels. Second, we categorize respondents into subsamples based on different levels of risk aversion and income in order to identify the differences in their valuations of food safety vs. quality labels. Third, we examine the impacts of loss aversion and income effect when making food choices based on food safety vs. quality labels. The results will provide valuable information to help the food industry decide whether to increase the publicity of safety or quality information labels, help distributors develop effective marketing strategies to meet consumer demand for food safety and quality, and assist policymakers in setting attribute priorities on food safety vs. quality when formulating food policies.

## Research Framework

### Research Hypotheses

Utility is a term in economics that refers to the total satisfaction or dissatisfaction that consumers experience when consuming a good or service. Economic theories based on rational choice usually assume that consumers will strive to maximize their utility, from the perspective of psychological expectations. Utility is also a subjective measure of satisfaction or dissatisfaction that varies from person to person according to each personal preference. Although consumer utility is impossible to quantify, utility losses or gains that a consumer obtains from different goods and services can be compared (Lancaster, 1966; Louviere et al., 2000). Consumers make food purchasing choices based on the comparison of self-assessed utility losses and gains in order to maximize their psychological expectations. The utility tradeoff between loss aversion and income effect, directly affected by each individual’s risk aversion and income level, determines whether a consumer will choose safe food that is above the minimum quality standard or high-quality food at a high price. Accordingly, we propose and validate hypothesis H1 about the heterogeneity in consumers’ valuations of food safety and quality labels.

H1: Consumers with varying levels of risk aversion and income place heterogeneous valuations for food safety and quality information labels.

**FIGURE 1:**
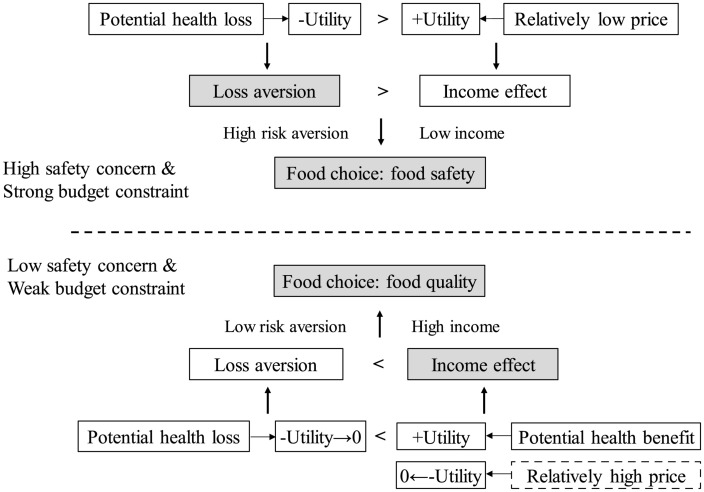
The influence of loss aversion and income effect on consumer food choice.

For consumers with a high safety concern and strong budget constraint, the potential health risk of consuming food without official or third-party safety-certified labels would significantly reduce consumer utility. At the same time, the cost savings that come with relatively low prices of safe food would increase consumer utility. Loss aversion, whereby the impact of losses overweighs gains, is typically examined in relation to decisions about anticipated outcomes (Boyce et al., 2013; Iwasaki et al., 2019; Koan et al., 2021). In general, probable utility losses in potential health risk have a larger negative impact on consumer well-being than equivalent utility gains in low food price. This implies that the negative utility caused by the insufficient food safety information would be larger than the positive utility created by the relatively low food price. Under this condition, the loss aversion for health risk would be amplified due to a high level of risk aversion and would outweigh the income effect created by low food prices. When consumers have a high level of risk aversion and a low level of income, they are likely to care more about the safety of what they eat than the quality of the food. These high risk-averse and low-income consumers tend to pay a premium for additional safety information and to choose safer food with safety-certified labels in order to reduce utility loss resulting from potential health risks. Accordingly, we propose and validate hypothesis H2 regarding the influence of loss aversion and income effect on consumers’ demand for food safety labels, as indicated by the upper half of Figure 1.

H2: When the utility losses from loss aversion outweighs the utility gains from income effect, consumers with high risk aversion and low income prefer safe food with safety information labels.

In another case, for consumers with low safety concern and weak budget constraint, the potential health benefit from consuming more nutritious and healthier food with higher quality would certainly increase consumer utility, while the relatively higher food prices would reduce consumer utility. However, because consumers have a low risk aversion for food safety, the loss aversion regarding potential health risks related to unsafe food would be weakened and the negative utility of loss aversion approaches 0. Similarly, the high price – which includes the labeling cost of high-quality food – has no apparent negative impact on high-income consumers’ utility. Here, the negative utility of income effect is also close to 0. In general, utility gains in health benefit have a larger positive impact on consumer well-being than the minimal utility losses in high food price and health risk. This implies that the positive utility created by high-quality food with sufficient quality information is the main factor driving low risk-averse and high-income consumers’ demand for food quality labels. Under this condition, the income effect for potential health benefit from high-quality food with high price far outweighs the loss aversion for potential health risk. Accordingly, we propose and validate hypothesis H3 about the influence of loss aversion and income effect on consumers’ valuations of food quality labels, as indicated by the bottom half of Figure 1.

H3: When the utility losses from loss aversion is less than the utility gains from income effect, consumers with low risk aversion and high income prefer high-quality food with quality information labels.

### Choice Experiment

Choice experiment is a quantitative technique that is used to elicit consumer preferences. It helps researchers to uncover how consumers value selected attributes of food products by asking them to state their preferences among a series of choice sets of hypothetical alternatives. Each alternative is described by several attributes, and consumers’ responses are used to infer the value placed on each attribute. Comparing with other standard contingent valuation techniques that require respondents to rank or rate alternatives, choice experiment presents a reasonably straightforward task, which more closely resembles a real-world decision. The theoretical foundation of choice experiment is conceptually based on Lancaster’s consumer utility theory, which postulates that consumers obtain utility from the attributes of a product rather than from the product itself (Lancaster, 1966).

Our choice experiment was carefully designed to specify five product attributes, including two food safety attributes (certification and traceability system), two food quality attributes (brand reputation and grading system), and the prices of rice products. The five specifications of food-specific attributes are summarized in Table 1. In our choice experiment, the rice products with safety-certified labels are mainly referred to as organic or green rice. Three cases of certification were considered; i.e., governmental certification label, third-party certification label, and no certification label. For simplicity, we considered two cases of traceability, brand, and grade: with and without traceability label, with and without brand, and with premium or standard grade. The traceability label in our survey is a QR code that can be scanned by cellphone to track food safety information about where the rice product comes from. Gold Arowana is a well-known rice brand in China and is highly regarded by consumers. Rice is graded into premium and standard grades mainly according to content of broken rice, length of rice, and milling degree, as specified in the National Standard Rice (No. GB 1354-2009). Brand is commonly specific to high value-added products, and by contrast, grading system applies to bulk agricultural products with low added values. The rice price takes three values, with a base price of 3 yuan/500g, a middle price of 5 yuan/500g, and a ceiling price of 7 yuan/500g.

**TABLE 1:** Information labels of rice products in choice experiment.

Information labels		Level 1	Level 2	Level 3
Food safety	(1) Certification	Governmental	Third-party	None
	(2) Traceability	QR code	No	
Food quality	(3) Brand	Gold Arowana	No	
	(4) Grade	Premium	Standard	
(5) Price (CNY/500g)		3	5	7

**FIGURE 2:**
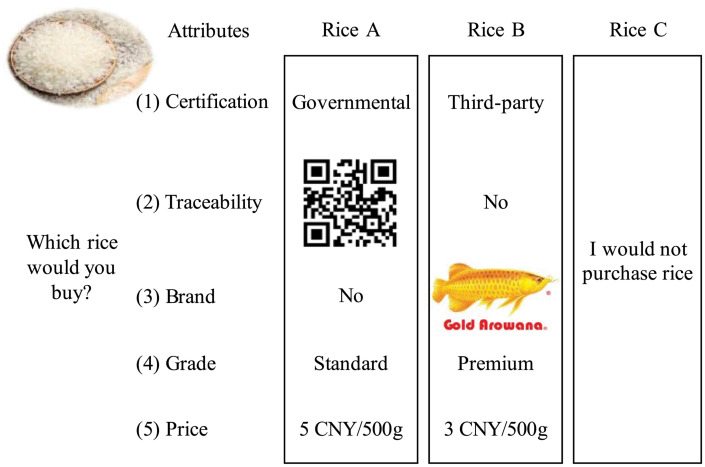
A rice choice set sample.

The five selected attributes can produce as many as 72 (3^2^ × 2^3^) combinations under a full factorial design. To minimize respondents’ response difficulties, a fractional factorial design orthogonally generated 16 combinations, each two of which were randomly split into one group. A respondent was presented with 8 simulated choice scenarios that each contained two alternatives characterized by the combinations of different levels of five attributes. The “do not buy” alternative was included to simulate a real shopping market where respondents were allowed not to purchase rice products. A choice scenario is illustrated in Figure 2.

Since the choice experiment does not provide incentives for respondents to invest sufficient cognitive effort when thinking about their valuation decisions, the WTP values may be overstated in the hypothetical situation because of the lack of real economic commitment. At the beginning of our survey, we use some *ex ante* methods to mitigate hypothetical bias. Specifically, hypothetical questions include the “do not buy” alternative (as an explicit option to adjust uncertainty), an objective cheap talk (designed to remind respondents to behave in the same way that you would if you really had to pay for the product), and honesty priming treatment (used to increase respondents’ honesty and to unconsciously manipulate their perception, appraisal, and behavior priming). Overall, de-Magistris et al. (2013) found some evidence that WTP values both in honesty priming treatment and cheap talk were the two closest to the WTP derived from non-hypothetical treatment. The hypothetical choice experiment, modified by these *ex ante* methods, would result in reasonable and robust valuation measures.

Our choice experiment was carried out across China’s western, middle, and eastern regions from May to July 2018. A group of well-trained enumerators were recruited to investigate three major capital cities: Chongqing (municipality), Changsha (capital of Hunan province) and Nanjing (capital of Jiangsu province), and six developing cites: Xinyang (Henan province), Luan and Bengbu (Anhui province), Xuzhou, Changzhou and Suzhou (Jiangsu province). These nine cities covered in our survey were selected from major Chinese rice consumption areas. In all, approximately 41% of experimental observations were derived from fresh markets, 46% from domestic supermarkets, and 13% from international supermarkets. This was done to better capture the heterogeneity of consumers’ purchasing behaviors in various purchasing contexts.

### Data Description

Table 2 shows the summary statistics based on selected demographic information from consumer surveys. The average age of the sampled respondent is 40 years old and acts as a primary shopper for her three family members. About 54 percent of respondents acquire a 4-year college degree. Nearly 60 percent of respondents’ household income level is between 6000 CNY to 15000 CNY. Furthermore, consumers’ perception of food safety risk and their attitude toward food safety risk are captured with the five-point Likert scales. On a 1-to-5 scale the median average risk perception is approximately 2.43, similar to the value reported by Lim et al. (2014). Respondents have a negative attitude about consuming food without safety labels, with an average risk attitude of 3.84 on 1-to-5 scale. A majority of respondents believe that the local regulatory system is efficient, and they have high confidence in the authenticity of food inspection information disclosed by the government.

**TABLE 2:** Socio-demographic statistics.

Variable	Description	Mean (SD)
Age	Years	39.64 (15.08)
Household size	Persons	3.13 (1.30)
Gender	Female = 1, male = 0	0.62 (0.49)
Child	Child in household = 1, No child = 0	0.42 (0.49)
Shopper	Chief shopper in household = 1, Not = 0	0.60 (0.50)
Education	Junior school or below = 1	13.01%
	Senior school = 2	23.79%
	College graduate = 3	53.91%
	Graduate degree = 4	9.29%
Monthly family Income	Less than 3,000 = 1	4.83%
(unit: yuan)	3,000–6,000 = 2	18.77%
	6,000–10,000 = 3	33.84%
	10,000–15,000 = 4	23.98%
	15,000–20,000 = 5	10.22%
	More than 20,000 = 6	8.36%
Risk perception ^1^	Strongly agree = 1, …, Strongly disagree = 5	2.43 (0.99)
Risk attitude ^2^	Strongly willing = 1, …, Strongly unwilling = 5	3.84 (1.42)

*China census data is the weighted mean based on 1% National Population Sample Survey 2015 and China Statistical Yearbook 2017. ^1^ Consuming rice without safety-certified labels is not risky at all. ^2^ You are strongly willing to accept the risk of consuming rice without safety-certified labels.*

## Materials and Methods

### Mixed Logit

Mixed logit is a highly flexible model that can approximate any random utility model (Mcfadden and Train, 2000). Generally speaking, it obviates the three limitations of standard logit by allowing for random taste variation, unrestricted substitution patterns, and correlation in unobserved factors over time. Following the consumer theory (Lancaster, 1966) and the random utility theory (McFadden, 1974), the utility that decision maker *n* obtains from choosing alternative *j* in choice situation *t* is given by

(1) $$U_{njt}=\beta_{n}^{\prime }x_{njt}+\varepsilon_{njt}$$

where *U*_*njt*_ is a function of observable attributes of the alternatives, *x*_*njt*_, and of the decision maker *n*. The random term μ_*njt*_ is assumed to be iid type I extreme value distributed. The mixed logit model extends the standard conditional logit model by allowing heterogeneous coefficients β_*n*_ in the model to vary across decision makers. The decision maker *n* knows the value of his own β_*n*_ for all alternatives *J* and will choose the alternative *i* that provides the highest level of utility, if and only if *U*_*nit*_ > *U*_*njt*_,∀*j* ≠ *i*.

To estimate consumers’ unobserved heterogeneous preferences for informational attributes, we select the mixed logit model, which is specified to include a combination of non-random coefficients and multivariate normal random coefficients (Hole, 2007). The mixed logit probability can be viewed as a weighted average of the logit formula evaluated over a density function of coefficients. By assuming that utility is linear in parameters β_*n*_, the choice probability of mixed logit model under density function *f*(β_*n*_) can be expressed in the form of

(2) $$P_{nit}=\int \frac{exp\left(\beta_{n}^{\prime }x_{njt}\right)}{\sum_{j=1}^{J}exp\left(\beta_{n}^{\prime }x_{njt}\right)}f\left(\beta_{n}\right)d\beta_{n}$$

### Willingness to Pay

The WTP for an attribute is interpreted as the level of compensation or discount for consumers relative to the utility without that attribute, which would be needed to make them indifferent to the two situations. To calculate mean WTP values for all consumers, a ratio is taken in which the numerator is the parameter on that attribute and the denominator is the negative price coefficient, which can be perceived as the marginal utility of money. The WTP for attribute *k* is represented by

(3) $$WTP_{k}=-\frac{\beta_{k}}{\beta_{price}}$$

where β_*k*_ and β_*price*_ are all decision makers’ mean coefficients for attribute *k* and for the price of rice product, respectively.

For the statistical properties of the WTP for attribute *k*, the Monte Carlo method developed by Krinsky and Robb (1986) is used to measure standard deviation and 95% confidence intervals. This simulation procedure of a parametric bootstrapping technique requires a large number of random draws (1000 draws in our case) for a parameter vector from a multivariate normal distribution utilizing a variance–covariance matrix and the means of estimated parameter vectors.

## Results and Discussion

Table 3 shows the mixed logit model estimation results for both the full sample and the subsample. To categorize consumers as low or high risk aversion, we aggregate the scores for each consumer, measuring individual risk perception and risk attitude as shown in Table 2. Consumers with a total value of 2 to 5 are categorized as “low risk aversion” subsample, while consumers with a total value of 6 to 10 are categorized as “high risk aversion” subsample. Similarly, the classification of consumers into the two groups of low and high income depends on their monthly family income. Consumers with monthly family income of less than 10000 CNY are classified as “low income” subsample, while consumers with an income of more than 10000 CNY are classified as “high income” subsample. Table 4 shows the mean values of consumers’ WTP for each food safety and quality label. Based on the mixed logit model estimation, each consumer’s WTP is derived from Krinsky and Robb (1986) parametric bootstrapping simulation by 1000 random draws for a parameter vector from a multivariate normal distribution and utilizing a variance-covariance matrix and the means of estimated parameter vectors.

**TABLE 3:** Results of the mixed logit model.

	Full sample	Low risk aversion	High risk aversion	Low income	High income
***Estimated coefficients in utility function***					
Price	−0.423***(0.020)	−0.417***(0.027)	−0.430***(0.031)	−0.469***(0.027)	−0.370***(0.032)
Governmental certification	1.072***(0.051)	0.945***(0.069)	1.225***(0.077)	1.038***(0.065)	1.149***(0.084)
Third-party certification	0.207***(0.045)	0.168***(0.060)	0.260***(0.071)	0.221***(0.058)	0.192***(0.075)
Traceability	0.771***(0.040)	0.615***(0.053)	0.959***(0.063)	0.783***(0.052)	0.769***(0.067)
Grade	0.677***(0.038)	0.578***(0.050)	0.807***(0.059)	0.609***(0.048)	0.793***(0.063)
Brand	0.752***(0.039)	0.600***(0.050)	0.927***(0.063)	0.721***(0.053)	0.807***(0.062)
Optout	−2.990***(0.126)	−3.011***(0.168)	−2.982***(0.194)	−2.962***(0.161)	−3.077***(0.207)
***Distributions of standard deviations of estimated coefficients***					
Governmental certification	0.373***(0.077)	0.437***(0.094)	0.213(0.173)	0.275**(0.126)	0.489***(0.107)
Third-party certification	−0.014(0.140)	0.012(0.141)	−0.143(0.298)	−0.012(0.153)	−0.104(0.370)
Traceability	0.297***(0.066)	0.298***(0.087)	−0.208(0.138)	0.210*(0.115)	−0.414***(0.085)
Grade	0.007(0.115)	−0.004(0.123)	0.017(0.261)	0.002(0.129)	0.035(0.164)
Brand	0.394***(0.053)	0.332***(0.077)	0.431***(0.077)	0.450***(0.066)	0.314***(0.091)
Optout	0.967***’(0.081)	0.884***(0.115)	1.102***(0.120)	0.959***(0.100)	0.874***(0.138)

Notes: ***, **, * denotes statistical significance at 1%, 5%, 10% levels, respectively. Standard errors are in parentheses.

**TABLE 4:** Estimated WTP (CNY/500g) of full sample and subsamples.

	Full sample	Low risk aversion	High risk aversion	Low income	High income
Governmental certification	2.532***	2.265***	2.846***	2.214***	3.102***
	[2.289, 2.782]	[1.947, 2.603]	[2.488, 3.247]	[1.957, 2.487]	[2.617, 3.667]
Third-party certification	0.488***	0.402***	0.605***	0.472***	0.519***
	[0.268, 0.720]	[0.107, 0.717]	[0.261, 0.988]	[0.215, 0.746]	[0.101, 0.986]
Traceability	1.821***	1.473***	2.228***	1.670***	2.077***
	[1.638, 2.023]	[1.246, 1.728]	[1.936, 2.568]	[1.468, 1.898]	[1.722, 2.499]
Grade	1.598***	1.384***	1.875***	1.299***	2.141***
	[1.436, 1.791]	[1.172, 1.638]	[1.634, 2.188]	[1.120, 1.518]	[1.839, 2.540]
Brand	1.776***	1.437***	2.154***	1.537***	2.180***
	[1.601, 1.987]	[1.223, 1.702]	[1.877, 2.511]	[1.340, 1.776]	[1.843, 2.621]

Notes: ***, **, * denotes statistical significance at 1%, 5%, 10% levels, respectively. 95% confidence intervals are in brackets.

For the analysis, the coefficients of food safety and quality labels were assumed as random and normally distributed. As shown in Table 3, the coefficients of price and optout for rice consumers are negative and significant, indicating that consumers are sensitive to rice price and that they would experience dissatisfaction from not buying the staple food. As expected, consumers strongly prefer food safety and quality labels that positively impact their utilities. Rice products with certificated, traceable, grade, and brand labels could provide detailed safety and quality information to help them make well-informed purchasing decisions and reduce the time cost of purchasing. As to the full sample, the first two columns of Table 3 indicate that the coefficients of three food safety attributes – governmental certification, third-party certification, and traceability – are significant and positive, which means consumers prefer rice with safety-certified and traceable labels. Consumers’ mean WTP values for price premium to achieve government-certified and traceable information are higher than third-party certification; however, as found in Table 4 the 1000 non-parametric bootstrap simulations show that consumer preferences for governmental certification and traceability are significantly heterogeneous while preferences for third-party certification is not. This implies that consumers are more divergent about the food safety labels on government-certified and traceable information, because they both guarantee a certain level of food safety. Moreover, consumers’ preferences for the other two food quality attributes, i.e., grade and brand, are also highly significant with similar magnitudes of estimated coefficients and WTP values, as indicated by Tables 3, 4. There exists heterogeneity in consumer preference for brand reputation, while they express more consensus about the role of quality grade label, which improves the level of food quality. The heterogeneities in the full sample’s valuations of food safety labels on governmental certification and traceability, as well as in their valuations of food quality labels on brand reputation, show a statistical significance of 1%, which validates the hypothesis H1 that was proposed in the second part of this paper. The next four columns of Tables 3, 4, respectively, compare the estimated results for the mixed logit model and WTP between various subsamples. While consumers’ preferences for food safety and quality labels are qualitatively similar across subsamples in terms of scales and directions (as evidenced by Table 3), the WTP values estimated in Table 4 notably differ compared to their preference coefficients.

The estimated WTP for food safety vs. quality labels within and between subsamples are compared in Table 5. The results are intuitive, showing that as consumers become increasingly concerned about the safety of food, and as their income grows, the WTP values either for food safety or quality labels both significantly increase. It implies that consumers with a higher income and a greater awareness of what they eat are more motivated to identify the level of food safety and quality. They are willing to pay a higher price for obtaining that inherent and unobvious food-related information. It shall be noted that whether consumers belonged to the high vs. low risk-averse group or the high vs. low income group, both groups were willing to pay a higher price for the cost of food safety labels than food quality labels, because food with a safety-certified information label can minimize consumers’ risk perception and risk attitude.

**TABLE 5:** WTP differences for food safety vs. quality labels within and between subsamples.

Group	High vs. low risk aversion			High vs. low income		
	I Low	II High	Δ High vs. low aversion: II-I	III Low	IV High	ΔHigh vs. low income: IV-III
(1) Food safety labels	1.729***	2.445***	0.716***	2.043***	2.110***	0.067***
(2) Food quality labels	1.178***	1.734***	0.557***	1.330***	1.599***	0.268***
ΔSafety vs. quality: (1)-(2)	0.552***	0.710***	0.159***	0.712***	0.511***	−0.201***

Notes: ***, **, * denotes statistical significance at 1%, 5%, 10% levels, respectively.

The WTP differences between subsamples are statistically significant, as the *t*-test statistics indicated in Table 5. Consumers in the high risk-averse group are willing to pay 0.716 CNY/500g more for food safety labels and 0.557 CNY/500g more for food quality labels than the low risk-averse group. The WTP difference value of 0.159 CNY/500g between safety and quality labels in the high vs. low risk-averse group implies that compared to low risk-averse consumers, food safety is considered a more preferable attribute for consumers who are highly risk-averse about the safety of what they eat. Conversely, consumers in the high-income group are willing to pay 0.067 CNY/500g more for food safety labels and 0.268 CNY/500g more for food quality labels than the low-income group. The WTP difference value of −0.201 CNY/500g between safety and. quality labels in the high vs. low-income group implies that compared to low-income consumers, high-income consumers give more attention to higher quality in more healthy and nutritious food that far exceed safety requirements.

Such WTP differences show that stronger loss aversion would produce a higher demand for food safety labels and a stronger income effect would produce a higher demand for food quality labels. The *t*-test statistics of WTP differences significantly validate the hypotheses H2 and H3 at the 1% level. It was expected that loss aversion and income effect would jointly influence differences in consumer reactions to the availability of food safety vs. quality labels. In terms of loss aversion, high risk-averse consumers experience a greater loss aversion when posed with potential health risks, making them more willing to pay a premium price to ensure that the rice product is safe enough. Low risk-averse consumers who present a weakening loss aversion correlated with psychologically perceived health risks tend to prefer food quality labels over food safety labels. In terms of income effect, high-income consumers show a strong willingness-to-pay when it comes to the cost of food quality labels in order to achieve more health benefits from high-quality food at a good price. For low-income consumers with a strong budget constraint, they are more likely to choose safe food that is above the minimum quality standard and with a relatively low price as opposed to higher priced high-quality food.

## Conclusion and Implication

China is now at a critical juncture in the transformation of food consumption and production. The decision on the part of public authorities and the food industry to prioritize food safety control or food quality improvement should center on market demand for either safe food that is above the minimum quality standard, or high-quality food held to a high quality standard. Although food safety and food quality are closely related in the food management system, there are significant differences between the two in that the former keeps consumers safe and the latter keeps consumers satisfied. However, to our knowledge there is no study in the current literature that integrates food safety and food quality into a comprehensive framework while evaluating the influence of consumers’ socio-demographic and psychological characteristics on preferences for food safety and food quality. Taking rice as a case study, this study employs choice experiment survey data and the mixed logit model in order to estimate the differences in consumers’ food choice for food safety vs. quality labels, and to uncover the influence of loss aversion and income effect on their valuations of food safety vs. quality labels.

The mixed logit model results show that Chinese consumers have heterogeneous preferences and WTP for a price premium to cover the cost of food safety and quality labels, with more divergent valuations of food safety labels on governmental-certified and traceable information, and food quality label on brand reputation. The density estimates of WTP differences indicate that although high-risk averse consumers express a higher WTP both for food safety and quality labels than low risk-averse consumers, the density distributions of high-risk averse consumers’ WTP for food safety labels and that of low risk-averse consumers’ WTP for food quality labels both exhibit a high degree of convergence. Similarly, although high-income consumers have a nearly equivalent WTP for food safety labels as low-income consumers, they have a higher WTP for food quality labels on average.

The WTP differences between subsamples indicate that loss aversion would produce a high demand for food safety labels and that income effect would produce a high demand for food quality labels. It was expected that consumers with various socio-demographic and psychological characteristics would react differently to the availability of food safety vs. quality labels in the food market. For high risk-averse and low-income consumers, they experience a greater loss aversion regarding potential health risks and a weaker income effect for high food prices, so that they tend to pay a price premium for easy access to safety indications assuring the safety of food. For low risk-averse and high-income consumers, the weaker loss aversion and greater income effect would result in a stronger willingness-to-pay for food quality labels that will bring them many health benefits from high-quality food at a good price.

Overall, our findings indicate that consumers with varying levels of risk aversion and income would be influenced by the loss aversion and income effects on their food choice for food safety vs. quality labels. This finding strongly suggests that the Chinese government and food industry should implement a multilevel food labeling scheme that integrates food safety and quality management systems. From the perspective of public policy, although ensuring food safety and improving food quality are two different policy objectives, with the government and food industry as the driving forces behind each, respectively, the two objectives should coexist within the common framework of China’s food management system. The modified food labeling scheme implemented jointly by the government and food industry would provide consumers more useful information to help them identify the inherent food safety and quality attributes, which cannot be easily identified otherwise. Such a fully information-asymmetric market can facilitate well-informed food choices and maximize consumer welfare.

Ensuring food safety is an important task that forms an integral part of the national planning strategy for building a healthy China and enhancing people’s well-being. The primary purpose of governmental food management is to guarantee that the food available to the public on the market meets the minimum quality standards, which is now being achieved over the last decade through a variety of initiatives including a modified food labeling scheme, rigorous random food inspection, and enhanced penalty severity. As governmental regulatory capacity is continuously strengthened, food safety risk will be effectively kept to an acceptable level, and consumers’ safety concern and their loss aversion regarding potential health risks will be weakened in terms of psychological expectations. Meanwhile, with the rapidly rising income levels, the current food market and policy climate overly focused on food safety control would not provide more valuable food quality information, nor would it satisfy the growing market demand for high-quality food among high-income consumers. Thus, the original policy objective of prioritizing food safety control over food quality improvement should be revised accordingly to keep up with the changing market demand for food safety vs. food quality. On the contrary, if the revised policy objective of food quality improvement is placed ahead of food safety control, an overemphasis on food quality improvement by the food industry would not only result in a sufficient safe food supply that far exceeds safety requirements, it would also result in increased production costs, higher food prices, and heavy expenditure burdens on low-income consumers who can hardly afford high-quality food at good prices and have a higher demand for safe food with relatively low prices.

Therefore, in the transition process from safe to high-quality food production and consumption, the government and food industry should balance the tradeoff between food safety control and food quality improvement. A multilevel labeling system contributes to a good market containing different levels of food quality with competitive prices, where consumers with varying levels of risk aversion and income would make appropriate food choices for safe food or high-quality food. Our findings provide the government with an effective labeling strategy for ensuring the safety of public diet, especially for the health interest of low-income and high risk-concerned groups. It also provides policy incentives for producers, processors, and distributors to collaborate and create a quality-differentiated food market with comprehensive food information in order to enhance the food industry’s competitiveness and to meet heterogeneous market demand for food safety vs. food quality.

This study identifies the dual effects of loss aversion and income on consumer food choice for food safety and food quality, but it is unclear whether the effects of education, cognitive performance, and other variables, particularly those affecting consumers’ valuations and choices, are at least as important for their food choices. Because the costs of promoting a modified labeling system are partially covered by food prices, the level at which the price increase should be limited relative to the current price in order to gurantee consumers’ benefit is of interest for future research. Furthermore, a further study comparing consumers’ valuations of mandatory vs. voluntary labeling systems could also establish valuable findings.

## Data Availability Statement

The raw data supporting the conclusions of this article will be made available by the authors, without undue reservation.

## Ethics Statement

The studies involving human participants were reviewed and approved by the Nanjing Agricultural University. The patients/participants provided their written informed consent to participate in this study.

## Author Contributions

WN: conceptualization, methodology, writing original draft. HB: methodology and data collection. JL: manuscript preparation, supervision, and editing. TL: conceptualization, writing original draft and editing. All authors contributed to the article and approved the submitted version.

## Conflict of Interest

The authors declare that the research was conducted in the absence of any commercial or financial relationships that could be construed as a potential conflict of interest.
